# Elucidating proton-intercalation chemistries

**DOI:** 10.1093/nsr/nwaf099

**Published:** 2025-03-17

**Authors:** Haocheng Guo, Simon Fleischmann, Chuan Zhao

**Affiliations:** School of Chemistry, University of New South Wales, Sydney, Australia; Helmholtz Institute Ulm (HIU), Germany; Karlsruhe Institute of Technology (KIT), Germany; Helmholtz Institute Ulm (HIU), Germany; Karlsruhe Institute of Technology (KIT), Germany; School of Chemistry, University of New South Wales, Sydney, Australia

## Abstract

The ion intercalation is fundamental for various rechargeable batteries, and the emerging proton intercalation exhibits distinct behaviors from the metal cations. Herein, the underlying mechanisms and unique electrochemical characteristics are elucidated.

Ion intercalation is one of the fundamental reaction mechanisms for rechargeable batteries and the choice of ion charge carriers affects their performance. Among all possibilities, protons stand out as charge carriers due to the smallest ionic radius (at the picometer level) and the capability for fast transport in aqueous media via the unique Grotthuss conduction. As a result, batteries that are based on proton intercalation are intrinsically advantageous in electrochemical reversibility and kinetics, making them highly promising for high-rate and long-life applications, and they are rapidly emerging [1]. An ion intercalation reaction generally involves three main steps: (i) electrolyte ion migration towards the interface, (ii) charge transfer across the electrolyte/electrode interface and then (iii) solid-state diffusion of intercalants in the host electrode. However, the mechanisms that underlie proton intercalation are not yet understood because protons often exhibit exceptional and unconventional behaviors. Regarding proton intercalation, although the first and last steps have been relatively well described, the charge-transfer process remains ambiguous and is the focus of ongoing investigations.

Metal cations are the predominant charge carriers for various batteries and are typically solvated in liquid electrolytes. For instance, Li^+^ is centrally coordinated with a number of solvent molecules to form a primary solvation sheath into which certain salt anions could also be incorporated under certain conditions (e.g. in highly concentrated electrolytes). Ion solvation varies in dimension and dissociation energy, depending on the ionic radius and charge density of the central cations. In contrast, protons exhibit fundamentally different solvation behaviors, likely due to the extremely small size and consequent high charge density. In the common case of aqueous solutions, protons form dynamic complexes (hydroniums) and Zundel (H_5_O_2_^+^) and Eigen (H_9_O_4_^+^) cations are the most characteristic representatives among many hypotheses, which have been recently verified by experimental observations as [H_2_O…H…OH_2_]^+^ and [H_3_O^+^(…OH_2_)_3_], respectively [1]. Illustrations of these hydroniums are shown in Fig. 1. These two forms of hydronium readily interconvert, indicating that the commonly adopted abbreviation for hydronium (H_3_O^+^) may not represent a stable, isolated entity. These hydronium ions can migrate as whole entities, accounting for one form of proton transportation in electrolytes (vehicular mechanism) that is similar to the common situations of metal cations. Additionally, electrolyte protons are able to be characteristically translated in events of concerted cleavage and the formation of an H–O covalent bond and an H^+^…H_2_O hydrogen bond through the network of water molecules. This displacive transportation (feature 1), known as Grotthuss proton conduction, is characterized by low activation energy (<0.4 eV, feature 2) and the spontaneous dynamic generation of a series of Zundel and Eigen cations (feature 3) [1]. These features serve as the defining criteria for authentic ‘Grotthuss conduction’ (mediated by water), which is sometimes misinterpreted but can be extended to other systems, e.g. the displacive iodide charge conduction [2].

**Figure 1.**
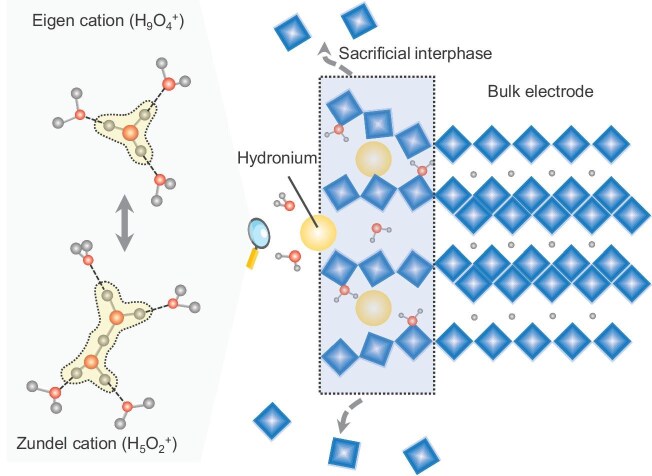
Facilitating hydronium desolvation and proton intercalation through a sacrificial interphase. The red and gray spheres represent oxygen and hydrogen atoms, respectively, while the cyan diamonds depict the structural units for the host electrodes. The yellow clouds highlight the cores of hydrated protons (hydroniums), which are simplified as yellow balls. The diagrams for hydroniums are adapted with permission [1]. Copyright 2023, John Wiley and Sons.

The charge-transfer step, which largely dominates the kinetics and stability of intercalation reactions, is particularly complicated for proton intercalation. In popular lithium-ion batteries, desolvation of coordinating solvents is a critical process that precedes naked-ion insertion into electrode lattices. This is facilitated by a solid–electrolyte interphase (SEI), which was first established during the development of graphite anodes via the electrolyte solvent replacement of propylene carbonate (PC) by mixtures of ethylene carbonate and other linear carbonate esters [3]. This substitution is important because the PC solvent has strong solvation capability and would co-intercalate with Li^+^ in the graphite lattice, causing its structure to irreversibly exfoliate due to the instable ternary intercalation compound. SEI layers are normally derived from the reduction of organic solvents and/or salt anions. Nevertheless, neither approach is applicable for proton batteries and a universal electrolyte-derived SEI has yet to be established. Furthermore, proton solvation structures are significantly different from those of Li^+^ and other metal cations, and hydroniums are reported to exhibit high desolvation energies. Therefore, one might naturally consider universal hydronium intercalation to occur, particularly given the good low-temperature performances that contrast with the sluggish desolvation that is typically observed in other systems. However, the reality turns out to be more complicated, or even the opposite.

Anomalies were first identified in α-MoO_3_, which is one of the most representative proton-intercalation electrodes. Investigations by Guo *et al.* [4] revealed dynamic mass evolution on electrodes by an electrochemical quartz crystal microbalance during proton intercalation, which is attributed to hydronium/water interactions (mass of H^+^ itself is negligible), whereas *operando* X-ray diffraction analysis indicates hydrogen molybdenum bronzes (H*_x_*MoO_3_) alone adequately account for all intercalation compounds, suggesting the occurrence of hydronium desolvation. Solid-state nuclear magnetic resonance (ssNMR) confirms desolvation by the absence of crystal water, but also detects confined water signals at the surfaces of materials with a disordered environment, which has yet to be fully understood. Additionally, a phenomenon of electrochemical dissolution is observed, serving as the major reason for electrode degradations. This phenomenon is intriguingly counterintuitive, as it is most pronounced in dilute aqueous acids but can be effectively suppressed in more concentrated aqueous acids (thereby excluding acidic corrosion) [4]. Subsequent investigations further reveal that the dissolution can be mitigated in other acidic electrolytes with reduced water fractions, in non-aqueous solvents [5,6] or even solvent-free electrolytes [7]. Moreover, recent studies also highlight the benefits of artificial ‘interphases’ in enhancing the kinetics and stability of electrodes by facilitating hydronium desolvation [6,8]. Notably, the same electrochemically driven dissolution phenomenon has also been identified across various proton-intercalation materials, including Prussian blue analogs [5], hydrous oxides (WO_3_·0.6H_2_O) [6], polyanion compounds (VPO_4_F) [7] and organic electrode materials (3,4,9,10-perylenetetracarboxylic dianhydride, PTCDA; pyrene-4,5,9,10-tetraone, PTO) [9,10], suggesting a universal mechanism underlying proton intercalation.

Reflecting on these insights, a theory that is analogous to SEI might help to explain the charge-transfer process during proton intercalation. Continuing with the representative α-MoO_3_, the inherent properties of hydronium drive a spontaneous trend for (partially) hydrated proton intercalation, which occurs initially at the surface lattices to form an interphase layer of transient ternary intercalation compounds (proton–water–host). This accounts for the detected surface water confinement and also aligns with the ultralow charge-transfer energy barrier (0.02 eV) reported for H_1.75_MoO_3_ electrodes (in 8.0 M H_2_SO_4_), which is attributed to a desolvation-free process [11]. As shown in Fig. 1, the interphase facilitates the proton transport while it suffers a mismatch with the bulk lattices, probably due to the significant variations in crystal structures, thus leading to gradual peeling (dissolution) and overall functioning as a sacrificial layer. This hypothesis may also be extended to hydrous host materials because the distinct spatial configurations of hydronium ions and free water molecules could pronouncedly influence the structure and internal electric fields. Conversely, it is also possible for solvated protons to intercalate in the bulk lattices of host electrodes, as evidenced by the direct hydronium insertion in tunnel-structured oxides [12]. On the other hand, the situations of organic electrode materials may differ from those of inorganic counterparts. Theoretical simulations in PTCDA offer a possible structure model that accommodates up to two H_3_O^+^ clusters [9]. This could be possibly attributed to the weak intermolecular interactions (e.g. van der Waals forces) of molecule crystals, allowing water molecules to enter deep into lattices, even in the presence of considerable electrochemical dissolution.

Regarding the solid-state diffusion of proton ions, recent studies have revealed that the characteristic Grotthuss conduction also operates in some hydrous host materials with percolating water networks, such as Prussian blue analogs [13], Ti₃C₂T_*x*_ MXenes [14], etc. It is noteworthy, however, that not all hydrous proton-intercalation electrode materials are applicable for Grotthuss conduction. For instance, studies on tungsten oxide hydrates (WO_3_·nH_2_O) reveal that [15], despite the abundant crystal water, the spatial configuration inside the crystal does not support the formation of Zundel/Eigen cations so Grotthuss conduction cannot work. Instead, proton transportation is found to proceed via a rotating–hopping mechanism through the lattice oxygen—a process that can also exhibit low activation energy like Grotthuss conduction. Proton-hopping transportation has been also identified in non-hydrous oxides (e.g. H_1.75_MoO_3_) [11] and is believed to be universal for oxide materials. Additionally, organic electrode materials that feature intramolecular hydrogen-bonding networks (e.g. hydroxyl group, amino moiety and crystal water) could achieve similar displacive proton transport in the Grotthuss manner [16]. These explored solid-state proton transportation mechanisms are different from those of metal cations, which vehicularly migrate within solid lattices. Notably, in emerging solvent co-intercalation chemistries, the transport behavior of solvated cations differs, as the coordinating solvents affect the ion migration within the host lattices [3]. Conversely, details regarding the solid-state diffusion of hydroniums or other proton–solvent complexes have yet to be reported.

Despite these advancements, a comprehensive understanding of the proton-intercalation process remains elusive. Current studies predominantly focus on reactions in Arrhenius (aqueous) acids at relatively high concentrations whereas alternative situations remain largely unexplored. For instance, certain material classes, such as manganese and vanadium oxides, also demonstrate considerable proton-intercalation capability but exhibit limited resistance to acidic corrosion compared with previously discussed materials. In these cases, proton intercalation often occurs in Lewis acids (e.g. aqueous solutions of Zn^2+^, Al^3+^ salts), possibly alongside other cations [1]. Although the phenomena have been recognized for some time, their underlying mechanisms are often oversimplified. Another critical gap lies in the context of solid electrolytes in which the nature of the ‘electrolyte proton’ may differ substantially, thereby influencing the entire intercalation process. Overall, proton chemistry and the associated intercalation reactions hold great promise for future battery applications. A clear understanding of the fundamental processes is essential to fully exploit the advantages of proton-based energy-storage systems.
